# Novel Roles of Non-Coding RNAs in Opioid Signaling and Cardioprotection

**DOI:** 10.3390/ncrna4030022

**Published:** 2018-09-17

**Authors:** Zesergio Melo, Cecilia Ishida, Maria de la Paz Goldaraz, Rocio Rojo, Raquel Echavarria

**Affiliations:** 1 CONACyT-Centro de Investigacion Biomedica de Occidente, Instituto Mexicano del Seguro Social, Sierra Mojada #800 Col. Independencia, Guadalajara 44340, Jalisco, Mexico; zcmelo@conacyt.mx; 2 Programa de Genomica Computacional, Centro de Ciencias Genomicas, Universidad Nacional Autonoma de Mexico, Cuernavaca 62210, Morelos, Mexico; ishida.cecilia@gmail.com; 3 Departamento de Anestesiologia, Hospital de Especialidades UMAE CMNO, Instituto Mexicano del Seguro Social, Guadalajara 44340, Jalisco, Mexico; dragoldaraz@hotmail.com (M.d.l.P.G.); rocioazulcielo@hotmail.com (R.R.)

**Keywords:** cardiovascular disease, cardioprotection, opioids, ncRNA, miRNA, lncRNA

## Abstract

Cardiovascular disease (CVD) is a significant cause of morbidity and mortality across the world. A large proportion of CVD deaths are secondary to coronary artery disease (CAD) and myocardial infarction (MI). Even though prevention is the best strategy to reduce risk factors associated with MI, the use of cardioprotective interventions aimed at improving patient outcomes is of great interest. Opioid conditioning has been shown to be effective in reducing myocardial ischemia-reperfusion injury (IRI) and cardiomyocyte death. However, the molecular mechanisms behind these effects are under investigation and could provide the basis for the development of novel therapeutic approaches in the treatment of CVD. Non-coding RNAs (ncRNAs), which are functional RNA molecules that do not translate into proteins, are critical modulators of cardiac gene expression during heart development and disease. Moreover, ncRNAs such as microRNAs (miRNAs) and long non-coding RNAs (lncRNAs) are known to be induced by opioid receptor activation and regulate opioid signaling pathways. Recent advances in experimental and computational tools have accelerated the discovery and functional characterization of ncRNAs. In this study, we review the current understanding of the role of ncRNAs in opioid signaling and opioid-induced cardioprotection.

## 1. Introduction

According to data from the Global, Regional, and National Burden of Cardiovascular Diseases study, more than 17 million people died globally from cardiovascular disease (CVD) in 2015 and a large percentage of these deaths were secondary to coronary artery disease (CAD) [1]. The presence of cholesterol plaques and inflammation in the coronary arteries that supply the heart with blood, oxygen, and nutrients is characteristic of CAD pathophysiology. Plaque growth progressively restricts blood flow in the arteries, which can result in ischemia-reperfusion injury (IRI) and myocardial infarction (MI) [2].

Even though prevention is the best strategy to reduce risk factors associated with MI, interventions such as thrombolytic therapy and primary percutaneous coronary intervention are currently being used to reduce myocardial ischemic injury and limit infarct size in patients with MI [3]. However, the development of other cardioprotective strategies able to further improve patient outcomes is of major interest. Opioid conditioning is an effective intervention to ameliorate myocardial damage in animal models and human subjects [4,5]. It involves the activation of opioid receptor signaling pathways and mimics some of the protective effects achieved by ischemic preconditioning (IPC) [6,7,8].

In recent years, non-coding RNAs (ncRNAs) such as microRNAs (miRNAs) and long non-coding RNAs (lncRNAs) have gained attention for their remarkable role in cellular function and disease development. The non-coding transcriptome is numerous, which is shown by the latest version of the human lncRNA collection NONCODE that contains 96,308 lncRNA gene loci and exceeds the 22,210 human genes annotated as protein-coding across the Ensembl/GENCODE, RefSeq, and UniProtKB databases [9,10]. Non-coding RNAs participate in DNA synthesis, maintain genome stability, and promote epigenetic modifications [11,12,13]. Most ncRNAs assemble RNA-protein complexes or exploit their intrinsic ability of base pairing to selectively bind and, thus, modulate proteins and other nucleic acids. Multiple studies have identified several ncRNAs implicated in cardiac gene expression and cardiomyocyte behavior during heart development and disease [14]. Furthermore, miRNAs and lncRNAs are known to be induced by opioid receptor activation and can regulate opioid functions in a variety of conditions [15,16]. Thus, polymorphisms affecting the function of ncRNAs involved in opioid signaling could also have a profound impact on drug behavior and efficacy. In this paper, we will summarize the current understanding of the role of miRNAs and lncRNAs in opioid signaling and opioid-induced cardioprotection.

## 2. The Opioid System

Opioids have potent analgesic effects and are routinely used as medications for managing peri-operative and post-operative pain [17]. Opioid receptor activation mediates the effects of endogenous and exogenous opioid ligands. The distribution of these receptors is broad and includes the central nervous system (CNS), peripheral sensory and autonomic nerves, and multiple cell types including cardiac cells. Activation of opioid receptors at pain modulating sites as well as in limbic, midbrain, and cortical structures prevents spinal cord pain transmission through neuron inhibition [18]. Additionally, the existence of opioid receptors throughout the body contributes to the regulation of complex behaviors and functions such as stress responses, reward processing, mood, respiration, endocrine changes, and immunity [19,20,21].

Opioid receptors belong to the G protein-coupled receptor (GPCR) superfamily. This family includes classical opioid receptors sensitive to naloxone and classified as delta (δ, DOR), kappa (k, KOR), and mu (μ, MOR) as well as the non-classical, naloxone insensitive nociceptin/orphanin receptor [22]. In 1973, three different research groups discovered the existence of opioid receptors in brain homogenates using opioid radio ligand binding [23,24,25]. The *Oprm1*, *Oprd1*, and *Oprk1* genes were first cloned in the early 1990s and encode MOR, DOR, and KOR, respectively [26,27,28,29]. Both exogenous alkaloids and endogenous peptides such as enkephalins, dynorphins, and β-endorphins can bind with variable affinity and activate opioid receptors. Morphine was the first opiate to be synthesized and is considered a classic MOR agonist [22]. The use of morphine and MOR-selective synthetic opiates called 4-anilidopiperidines, which include fentanyl, remifentanil, sufentanil, alfentanil, lofentanil, ohmefentanil, and carfentanil is common in clinical practice. The potency of these synthetic opiates is between 100-fold and 10,000-fold higher than morphine [30].

Opioid receptors belong to the G protein-coupled receptor (GPCR) superfamily, which is a class of receptors that signal through intracellular heterotrimeric G proteins [18]. Upon activation with opioid agonists, guanosine diphosphate (GDP) is exchanged for guanosine triphosphate (GTP) on the α subunit of heterotrimeric G proteins, which is followed by the dissociation of α and βγ subunits. This dissociation leads to the subsequent interaction of α and βγ subunits with multiple downstream effectors including adenylyl cyclase, phospholipase C, protein kinase C (PKC), and ion channels. All four opioid receptors couple to pertussis toxin-sensitive G proteins like Gαi to inhibit adenylyl cyclase and cyclic adenosine monophosphate (cAMP) formation. Gα subunits can also interact directly with inward rectifying potassium (K^+^) channels considered essential regulators of electrical excitability in cardiomyocytes [31]. Opioid receptors are also known to interact with and modulate calcium (Ca^2+^) channels. Thus, when activated they reduce Ca^2+^ currents by closing P/Q-type, N-type, and L-type voltage-gated Ca^2+^ channels [32].

Additionally, opioid receptor activation initiates a non-canonical signaling pathway dependent on GPCR kinases (GRKs) and β-arrestins to induce receptor internalization and desensitization [33]. Recycling or degradation of opioid receptors occurs when GRKs trigger signaling termination through opioid receptor phosphorylation, which is followed by recruitment of β-arrestins and formation of receptor complexes able to internalize via clathrin-coated pits [34]. B-arrestins can also act as adaptors to recruit a wide range of signaling molecules to opioid receptor complexes including Src and activate downstream effectors such as mitogen-activated protein kinases (MAPKs) [35].

Opioid signaling via GPCRs can be agonist-selective and, as such, agonists that bind to the same receptor can induce different physiological responses by activating specific downstream signaling pathways [36]. Agonist-selective opioid signaling differentially modulates gene expression, which partially explains some of the conflicting results obtained with different agonists regarding cardioprotection and might have significant implications for opioid tolerance [37,38,39]. During opioid signaling, β-arrestin plays a significant role in the intracellular trafficking of GPCRs and β-arrestins bound to GPCRs also function as signal transducers of MAPKs and the serine/threonine kinase Akt [40]. Opioid agonists can induce extracellular signal-regulated kinase (ERK) phosphorylation through PKC or the β-arrestin pathway [41,42,43]. Morphine and fentanyl can both activate MOR but they differentially phosphorylate ERK [38]. ERK phosphorylation by morphine requires PKC activation and, in this case, ERK remains cytosolic and activates p90 ribosomal S6 kinase [37]. However, morphine ability to induce receptor phosphorylation, β-arrestin recruitment, and receptor internalization is limited in comparison to fentanyl [44]. ERK phosphorylation in response to fentanyl occurs in a β-arrestin dependent manner and leads to ERK translocation into the nucleus where it activates the transcription factor E26 transformation specific ETS domain-containing protein (ELK1) [38,44]. Thus, the differential modulation of opioid receptor trafficking and desensitization is due to the different ability of morphine and fentanyl to activate the β-arrestin pathway.

## 3. Mechanisms of Opioid Conditioning in the Heart

In CAD, blood flow disruption to the myocardium blocks the oxygen supply, which causes a rapid decline in ATP levels and an increase in AMP/ATP ratios [45]. The cardiac action potential shortens and within minutes of the occlusion extracellular accumulation of K^+^ occurs partly due to the opening of ATP-dependent K^+^ channels (K_ATP_) that generate an outward K^+^ current [46]. Since oxidative phosphorylation supplies most of the energy required for cardiomyocyte function, a lack of oxygen and nutrients unleashes profound metabolic changes within the affected cells. These changes include the arrest of oxidative phosphorylation, membrane depolarization, ATP depletion, 3Na^+^-2K^+^ ATPase dysfunction, intracellular sodium (Na^+^) overload, inhibition of contractile function, and a switch to anaerobic glycolysis [3,47]. Consequently, the cells experience lactate buildup, intracellular pH reduction, activation of the Na^+^-H^+^ ion exchanger, and Ca^2+^ overload. Thus, the ischemic death of cardiomyocytes begins in the subendocardial myocardium and, when the ischemia is prolonged, it extends transmurally across the ventricular wall and towards the epicardium, which causes irreversible damage [48]. There are reports of cardiomyocytes suffering from apoptosis, necrosis, and autophagy in response to ischemic insults [49]. Although reperfusion of acutely ischemic myocardium restores blood flow and normoxia, the events that accompany reperfusion can independently induce cardiomyocyte death, arrhythmias, myocardial stunning, and microvascular obstruction [50,51].

In 1986, Murry, Jennings, and Reimer described how several brief periods of coronary artery occlusion and reperfusion before a prolonged ischemic insult reduce ATP depletion and tissue damage [52]. This intervention is known as IPC. Several endogenous and exogenous mediators such as adenosine, bradykinin, acetylcholine, angiotensin II, endothelin, and opioids activate membrane-bound GPCRs on cardiac cells to mimic IPC [8,53,54,55]. Administration of morphine, remifentanil, methadone or U50488H, a KOR agonist, before ischemia or during reperfusion protects from myocardial damage in animal models [8,56,57]. Furthermore, evidence from multiple studies demonstrates that opioid receptor antagonists block IPC. Naloxone, which is a non-selective opioid receptor antagonist, as well as selective antagonists such as DOR and KOR prevent IPC in multiple species [58,59]. Moreover, δ1-opioid receptor/K_ATP_ channel signaling pathway mediates the cardioprotective effects of morphine and PKCδ is an essential mediator of infarct size reduction after δ1-opioid receptor stimulation [60]. Multiple signaling pathways activated in cardiomyocytes downstream of opioid receptors are known to be involved in opioid-induced cardioprotection. Some of these pathways include PKC, MAPKs (ERK1/2, p38, and JNK1/2), mammalian target of rapamycin, cyclooxygenase 2, inducible nitric oxide synthase, K_ATP_ channels, and reactive oxygen species [60,61,62,63,64,65,66,67]. Additionally, opioid receptor agonists such as piritramide, pethidine, fentanyl, sufentanil, carfentanil, and buprenorphine elicit arrhythmogenic protection in animal models of acute coronary artery occlusion [68,69,70]. Opioid-induced tolerance to arrhythmia during myocardial ischemia appears to be mediated by the activation of peripheral δ2-opioid and κ1-opioid receptors [71].

Research on opioid receptor activation in the adult myocardium has focused mainly on DOR and KOR while the contributions of MOR have mainly been ignored due to its low expression in healthy cardiac tissue [72,73,74]. However, MOR expression and its ligand-binding activity increase after ligation of the coronary artery in animal models and in isolated failing hearts [75,76]. Morphine or remifentanil administration before myocardial IRI induces MOR-mediated protection in failing hearts but not in healthy ones [76]. These protective effects appear to be dependent on phosphorylation of ERK1/2 and glycogen synthase kinase 3-βdownstream of MOR activation. Thus, MOR is a potential therapeutic target for opioid-induced cardioprotection during heart failure.

## 4. Non-Coding RNAs

**Figure 1:**
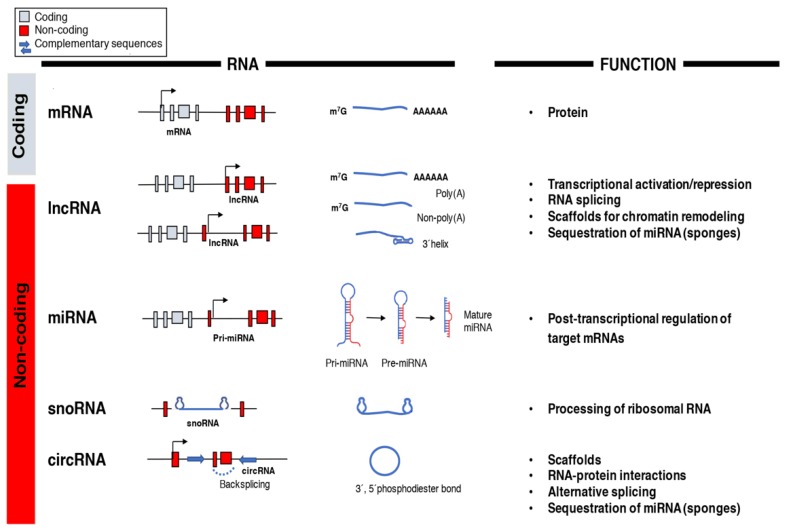
Classification and function of coding and non-coding RNAs (ncRNAs). The array of RNA molecules is diverse in structure and function across species. Messenger RNAs (mRNAs) are coding transcripts that undergo extensive post-transcriptional processing, 5′capping (m^7^G) and 3′polyadenylation (poly(A)), and can be translated into proteins. Long non-coding RNAs (lncRNAs) possess little, if any, coding potential and, after their transcription from genic and intergenic regions, they can suffer different types of post-transcriptional processing. MicroRNAs (miRNAs) are transcribed into primary miRNA transcripts (pri-miRNA) by Pol II or are generated through alternative splicing. They are further processed into pre-miRNA and mature miRNA before being incorporated into the RNA-induced silencing complex (RISC)to silence their target genes. Small nucleolar RNAs (snoRNAs) are usually transcribed by Pol II and belong to the translational machinery of the cell and process ribosomal RNAs. Circular RNAs (circRNAs) are produced from back splicing of exons and possess unique features given by the formation of a 3′,5′phosphodiester bond.

The central dogma of molecular biology established a directional flow of genetic information from DNA to RNA to protein and was still a prevailing idea in the 1970s [77]. It was only after the human genome was decoded in the post-genomic era that the real potential of RNA molecules other than messenger RNA (mRNA) as functional regulators of gene expression and cellular functions began to be appreciated [11]. The arrival of next-generation sequencing led to the discovery of multiple RNA species like miRNAs, lncRNAs, small nucleolar RNAs (snoRNAs), and circular RNAs (circRNAs) with unique biochemical and structural features whose functions are still not entirely understood (Figure 1) [78]. However, recent data highlights essential roles of the non-coding transcriptome in cell biology and human disease particularly in the regulation of gene networks through epigenetic modifications, direct transcriptional regulation, RNA processing, and inhibition of translation [79,80].

The first miRNA genes known as *lin-4* and *let-7* and their role as post-transcriptional regulators of gene expression were described in *Caenorhabditis elegans* during the early 1990s [81,82]. Micro RNAs are small, ncRNA molecules of ~22 nucleotides in length that use short regions of complementarity of around six to eight nucleotides of their target mRNAs to repress gene expression [83,84]. Endogenous miRNA-complementary sites are located within the 3′ and 5′ untranslated regions (3′ and 5′ UTRs) of target mRNAs as well as in the coding sequence [85,86,87]. Moreover, combinatory interactions between miRNA and both 3′ and 5′ UTRs of an mRNA have also been reported [88]. Biogenesis of miRNAs begins in the nucleus where RNA polymerases (Pol) II or III transcribe miRNA genes into primary miRNA transcripts (pri-miRNA) [89,90]. Then a nuclear microprocessor complex comprised of the nuclear RNase III enzyme Drosha and DiGeorge critical region 8 protein cleave pri-miRNAs to generate a precursor miRNA (pre-miRNA) [91,92]. Drosha-mediated processing of pri-miRNAs into pre-miRNAs is not required when miRNAs are derived from introns that have been released from their transcripts by alternative splicing [93]. Pre-miRNAs exported into the cytoplasm by exportin 5 are then processed by a RNAse III enzyme, which is called Dicer to generate a short RNA duplex that will give origin to a mature, single-stranded miRNA of around 20 to 30 nucleotides in length [94,95,96,97]. Two different mature miRNAs can arise from an miRNA duplex (-3p and -5p) but usually only one strand is incorporated into the RNA-induced silencing complex to guide the repression of its target mRNAs [98]. When the base pairing between miRNAs and their mRNA targets is fully complementary, it leads to direct cleavage and degradation of the target mRNA through a process involving the machinery of RNA interference while, if miRNAs are only partially complementary to their mRNA targets, the stability of the mRNA target is maintained but the result is inhibition of protein synthesis [89,90].

LncRNAs are a diverse group of long RNA transcripts (>200 nucleotides) without a protein-coding role and, unlike mRNAs, exhibit poor primary sequence conservation, tend to be shorter, and are usually expressed at relatively low levels [99]. This classification separates lncRNAs from short ncRNAs such as tRNAs, miRNAs, and snoRNAs and from longer, protein-coding transcripts. RNA polymerase II transcribes lncRNAs from different DNA elements (promoters, enhancers, and intergenic regions) [100,101]. LncRNAs usually suffer extensive post-transcriptional processing to reach their mature forms. This processing usually involves 5′-capping, splicing, and polyadenylation even though long primary transcripts can also generate mature lncRNAs without 5′ cap structures or 3′ polyadenylated tails (Figure 1) [101]. Some of the non-canonical mechanisms of lncRNAs processing include ribonuclease P cleavage to generate mature 3′ ends, which caps at their ends by snoRNA–protein complexes or circular structure formation [79,101]. Although lncRNAs are considered non-coding transcripts, some contain cryptic open reading frames [102]. LncRNAs act as scaffolds by forming RNA–protein complexes, perform decoy functions by interacting with DNA-binding proteins like transcription factors, work as enhancers in *cis* to activate or repress genes, and exert epigenetic regulation when they recruit chromatin-modifying proteins to specific genomic sites [103,104]. Additionally, lncRNAs exhibit a diverse repertoire across species and possess nuclear roles as well as cytoplasmic functions [105]. Competing endogenous RNAs (ceRNAs), which contain miRNA-binding sites and act as miRNA sponges, are an example of lncRNAs with cytoplasmic functions [106,107].

## 5. miRNAs in Control of Opioid Signaling

Several ncRNAs are known to be under the control of opioid receptors and can act as fine tuners of opioid signaling pathways by working as downstream targets of GPCRs and associated signaling molecules [108]. Evidence suggests that miRNAs can have profound effects on opioid receptor expression and, thus, in opioid receptor-mediated physiological and pathological processes. A genome-wide association study in human monocyte-derived macrophages treated with morphine identified 26 differentially expressed miRNAs (Table 1) of which miR-15b-5p and miR-181b-5p participate in inflammatory and oxidative stress processes responsible for human immunodeficiency virus 1 (HIV1) viral reservoir expansion in the CNS [109]. According to these results, opiates promote HIV1 propagation in immune cells while simultaneously suppressing immune functions. In a different study, oral administration of hydromorphone and oxycodone induced differential changes in plasma profiles of 179 miRNAs [15]. These opioids upregulate 9 miRNAs and downregulate 17 others (Table 1). Many of these miRNAs especially the let-7 family of miRNAs, miR-23b-5p, and miR-16-5p, have been associated with MOR signaling regulation elsewhere [110,111,112].

The 3′ UTRs of MOR, KOR, and DOR mRNAs contain multiple regulatory elements that are able to modulate their translational efficiency, mRNA stability, and transport [118,119]. Evidence indicates that opioids affect MOR expression through alternative splicing and changes in translational efficiency via miRNAs rather than altering its transcription [120,121]. The let-7 family of miRNAs, which include miR-23b-5p, miR-212/132, miR-16-5p, miR-339-3p, and miR-103/107, have all been identified as miRNAs that are able to bind to MOR 3′ UTR to post-transcriptionally repress its expression.

The let-7 family of miRNAs is abundant across species and its members share a ‘seed sequence’ responsible for mRNA target recognition by the RNA-induced silencing complex (RISC), which suggests that their functions are evolutionarily conserved and highly redundant [122,123]. Twelve different genomic loci in the human genome with some clustered together encode the let-7 family of miRNAs [124].

Although the expression of let-7 miRNAs is ubiquitous and appears to be context-dependent, it is clear that they are essential regulators of proliferation, stem-cell renewal, and terminal differentiation [125,126,127,128]. Interestingly, the expression of let-7 miRNAs increases in response to morphine treatment in neuroblastoma derived SH-SY5Y cells and these miRNAs modulate opioid tolerance by post-transcriptionally regulating MOR expression [110]. Thus, MOR downregulation represents one of many mechanisms contributing to opioid tolerance. In multiple studies, MOR mRNA expression remains unaltered by opioid agonists, which suggests that agonist-selective MOR downregulation occurs post-transcriptionally [129,130,131,132,133]. Let-7 miRNAs can downregulate MOR without affecting its mRNA expression through a mechanism that interferes with translation initiation [134]. Translational repression occurs when cytosolic let-7 miRNAs get incorporated into RISC and its interaction with MOR 3′ UTR leads to MOR mRNA recruitment to P-bodies where it becomes degraded by de-capping enzymes and exonucleases [110,134]. Other mechanisms are likely involved in opioid tolerance besides let-7 downregulation of MOR since knocking down let-7 in the brain only partially reduces morphine antinociceptive tolerance.

Wu Q et al. first described miR-23b-5p as a repressor of MOR translation through its interaction with a K box motif located in MOR 3′ UTR [113]. miR-23b-5p inhibits the association of MOR mRNA with polysomes and is responsible for suppressing MOR translation in mouse neuronal N2A-MOR cells. The same group later demonstrated that long-term morphine treatment upregulates miR-23b-5p in a dose-dependent and time-dependent manner, which describes miR-23b-5p as a post-transcriptional negative feedback mechanism between MOR activation and MOR expression [111].

Expression of the miR-212/132 cluster is required for adequate development, maturation, and function of neurons [135]. Morphine regulates the miR-212/132 cluster via MOR activation and its expression actively represses MOR mRNA translation, which highlights the close relationship that exists between these miRNAs and opioid signaling [114]. Transcription and processing of miR-212/132 occur through the activation of the MAPK MEK1/2, calmodulin, calmodulin kinases II and IV, and protein kinase A that converge in phosphorylation of cAMP Response Element Binding (CREB) protein. Additionally, the miR-212/132 cluster promoter is regulated by the methyl CpG binding protein 2, which forms an epigenetic protein complex and acts as a transcriptional repressor on methylated DNA after sustained morphine administration [114,136]. Another miRNA that post-transcriptionally targets MOR 3′ UTR is miR-134-5p. In a model of Freund’s adjuvant-induced chronic inflammatory pain downregulation of miR-134-5p inversely correlates with MOR expression in rat dorsal root ganglia and functional experiments revealed that MOR is a target of miR-134-5p [137]. Similarly, miR-339-3p expression increases in hippocampus isolated from mice chronically treated with opioid agonists [115]. Furthermore, morphine and fentanyl differentially modulate miR-339-3p expression levels through increased transcription of its pri-miRNA. Thus, the consistent differences between fentanyl and morphine in their ability to modulate miR-339-3p expression hint to molecular mechanisms underlying their distinct pharmacological properties.

The ubiquitous and highly conserved miR-16-5p is a miRNA linked to tumorigenesis, cell cycle regulation, proliferation, and apoptosis [138]. miR-16-5p also exerts miRNA-mediated post-transcriptional MOR regulation. Morphine inhibits miR-16-5p expression in lymphocytes, which is an effect that can be reversed by the antagonist naloxone [112]. Interestingly, miR-16-5p suppresses MOR expression when it binds to a site in its 3′ UTR located between 8699 and 8719 nucleotides away from the stop codon [112]. Thus, downregulation of miR-16-5p is a post-transcriptional mechanism through which morphine increases MOR receptor levels by stabilizing its mRNA.

**Figure 2:**
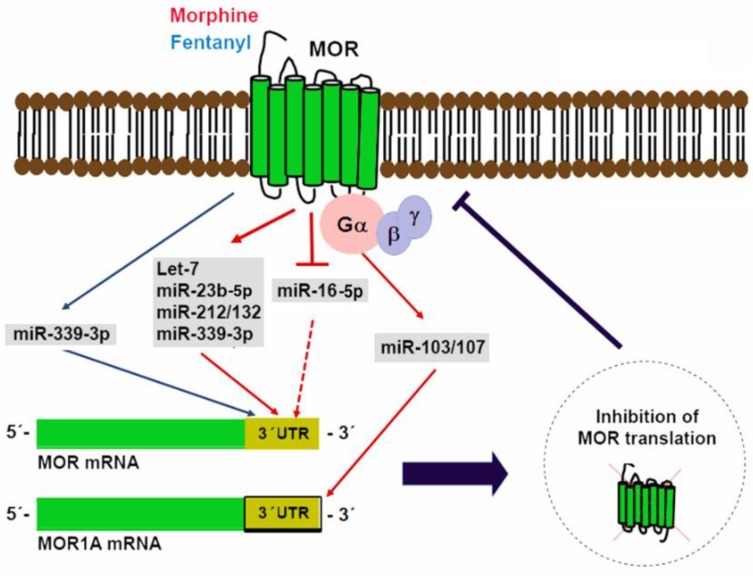
miRNAs in control of mu opioid receptor (MOR) translation. Multiple miRNAs able to bind to regulatory elements located in MOR 3′ UTR have been identified. Expression of some of these miRNAs (miR-339-3p, let-7, miR-23b-5p, miR-212/132, and miR-103/107) is induced by opioid agonists as a negative feedback mechanism. In contrast, morphine can also reduce miR-16-5p expression to increase MOR translation.

The *Oprm1* gene suffers considerable alternative splicing that results in the generation of multiple splice variants including a carboxyl-terminal splice variant that retains exon 3b and is known as MOR1A [116,139]. A conserved miR-103/107 targeting site in the 3′ UTR of both mouse and human MOR1A was identified through computational modeling and was further verified as a functional binding site for miR-103/107 in HEK-293 cells and a neuroblastoma cell line that endogenously expresses human MOR1A [116]. miR-103/107 is a pair of miRNAs with almost identical sequences except for a nucleotide located at the 3′-end and overlapping targets [140,141]. In a wide variety of tissues, the transcription of these miRNAs occurs from introns that map to three different pantothenate kinase family genes that encode regulatory enzymes necessary for coenzyme A biosynthesis [142,143]. In a neuroblastoma cell line and the striatum of a morphine-tolerant mouse, chronic morphine treatment increases miR-103/107 levels, which, in turn, reduce polyribosome-associated MOR1A mRNA [116]. Evidence that miR-103/107 regulate an *Oprm1* splice variant suggests that miRNAs could differentially regulate other opioid receptor variants with diverse 3′-UTRs. Figure 2 shows a graphical depiction of miRNAs in control of MOR translation.

Although fewer studies of DOR and KOR post-transcriptional regulation exist, DOR is a direct target of miR-874-5p in the context of hepatocellular carcinoma [144]. In this context, miR-874-5p expression inversely correlates with DOR expression and miR-874-5p downregulation is associated with a larger tumor size, increased vascular invasion, poor tumor differentiation, and worse outcomes.

In addition to miRNAs that regulate opioid receptor expression, miR-365-5p is a regulator of β-arrestin 2 able to reverse morphine anti-nociceptive tolerance in a rat model [117]. Neurons of the spinal cord express miR-365-5p and its levels decrease after chronic morphine administration. Thus, miR-365-5p plays an essential regulatory role in the development of morphine tolerance by targeting β-arrestin 2, which is a key molecule in opioid signaling and receptor trafficking [117,145].

## 6. Non-Coding RNAs in Opioid-Induced Cardioprotection

DOR activation protects against ischemic injury in the heart. Novel evidence demonstrates that DOR signaling affects miRNA expression in the heart and some of these miRNAs could be involved in mediating its protective effects (Table 2). Administration of the DOR agonist UFP-512 in rats maintained under normoxic conditions increases cardiac expression of miR-107-3p, miR-141-3p, and miR-350-5p while DOR activation under hypoxic conditions increases miR-7a/b, mi-107-3p, miR-200b-5p, miR-376a-3p, and miR134-5p levels [146]. Even though the exact contribution of these miRNAs and their targets to DOR-mediated cardioprotection is unknown, some of them are key modulators of apoptosis, angiogenesis, and tissue repair [147,148,149]. miR-107-3p targets hypoxia-inducible factor 1 βand prevents endothelial progenitor cell differentiation during hypoxia. This demonstrates the role of this miRNA in tissue repair during ischemic heart disease [147]. miR-200b-5p promotes angiogenesis by inhibiting its target ETS1 and miR-7a/b expression acts as a protective mechanism against IRI-induced apoptosis through poly(ADP-ribose) polymerase repression [148,149].

Additionally, morphine preconditioning modifies the miRNA expression profile in isolated cardiomyocytes while decreasing cell death and lactate dehydrogenase levels in models of hypoxia/reoxygenation in vitro [150,151]. A total of 39 miRNAs are differentially expressed in adult rat ventricular myocytes after morphine conditioning (Table 2) of which miR-133b-5p appears to have a preponderant role in morphine signaling and cardioprotection [150].

**Figure 3:**
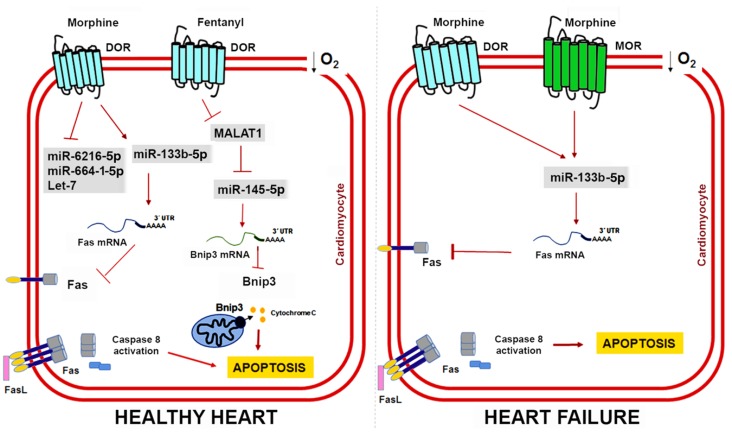
ncRNAs and opioid-induced cardioprotection in healthy and failing hearts. Experimental evidence shows that opioids can reduce cardiomyocyte apoptosis caused by myocardial IRI through miR-133b-5p and its target Fas in both healthy and failing hearts. In healthy hearts, fentanyl activation of DOR inhibits MALAT1 expression and a ceRNA for miR-145-5p, which allows miR-145-5p to target Bnip3 and reduce apoptosis. Interestingly, heart failure induces expression of MOR in cardiomyocytes, which could have important implications for opioid-mediated cardioprotection in patients with MI.

Initially defined as a muscle-specific miRNA, miR-133b belongs to a family of myomiRs that also includes miR-133a, miR-133b, miR-1, and miR-206. These miRNAs share tissue expression specificity but their mature sequences present nucleotide variants and, as such, their functions could be different [153]. Morphine administration in cardiac muscle cells upregulates miR-133b-5p expression and its knockdown blocks the protective effects of morphine preconditioning by promoting Fas expression, which is a death receptor that induces apoptosis by interacting with the Fas ligand [150]. Interestingly, some studies have described opioidergic conditioning interventions as being less effective in pathological conditions such as hypertrophy and heart failure [154,155,156]. To address this issue, Zhu HJ et al. further compared the expression profile of miRNAs in a rat model of heart failure induced by chronic doxorubicin injection [151]. They found 12 miRNAs whose expression pattern was significantly altered by hypoxia and morphine preconditioning (Table 2) and validated miR-133b-5p, miR-6216-5p, miR-664-1-5p, and let-7e as cardioprotective miRNAs in vitro. In cardiomyocytes derived from failing hearts, miR-133b-5p is severely downregulated, but morphine administration restores its expression [151]. Moreover, miR-133b-5p also mediates the protective effect of morphine in cardiomyocytes derived from failing hearts through its target Fas. However, the protective role of other miR-133b-5p targets remains to be determined. Morphine regulates the dopaminergic neuron differentiation in zebrafish via miR-133b-5p and its target Pitx3 and miR-133b-5p also targets the small GTPase RhoA to promote neurite outgrowth and spinal cord regeneration [157,158,159]. Figure 3 shows the cardioprotective mechanisms mediated by opioids and ncRNAs in healthy hearts and damaged hearts.

**Figure 4:**
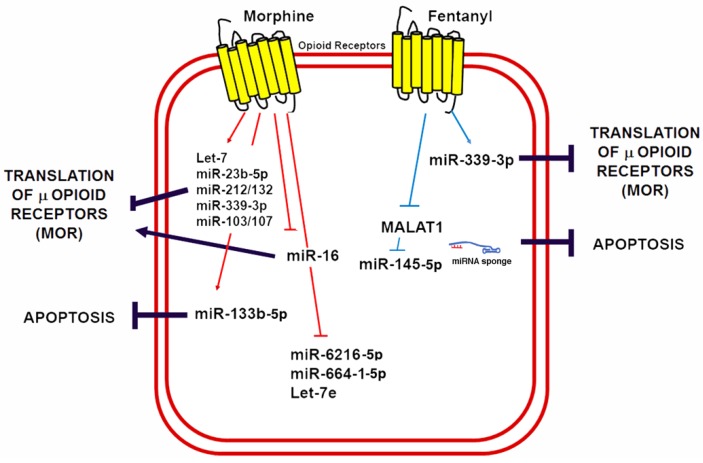
Morphine and fentanyl induction of ncRNAs involved in opioid signaling and cardioprotection. A schematic summary of miRNAs known to be induced by opioids in a variety of cells and animal models as well as its mechanisms of opioid signaling regulation and its potential implications for cardioprotection by reducing apoptosis.

Knowledge regarding the role of lncRNAs in opioid signaling and cardioprotection is still in its early days. Nonetheless, recent studies have demonstrated that lncRNAs could be a therapeutic target in myocardial IRI [160,161]. Metastasis-associated lung adenocarcinoma transcript 1 (MALAT1) is a lncRNA upregulated in response to hypoxia and myocardial ischemia [162,163]. Increased MALAT1 associates with cardiomyocyte apoptosis and its downregulation improves the ventricular function after myocardial IRI [164]. Moreover, fentanyl reduces hypoxia/reoxygenation injury through the MALAT1/miR-145-5p/Bcl-2 interacting protein 3 (BNIP3) axis [152]. MALAT1 promotes apoptosis by acting as a ceRNA for miR-145-5p, which allows the expression of miR-145 target BNIP3 (a pro-apoptotic protein associated with autophagy) [165]. Fentanyl exerts its protective effects through MALAT1 downregulation and negatively regulates the miR-145-5p/BNIP3 pathway in vitro and in mice subjected to myocardial IRI [152]. Figure 4 summarizes the role of ncRNAs in opioid signaling and opioid-induced cardioprotection.

## 7. Future Perspectives

Non-coding RNAs are essential modulators of opioid signaling and gene expression in physiological processes and CVD. A better understanding of the molecular networks regulated by miRNAs and lncRNAs in response to the activation of opioid signaling pathways in the heart may be exploited in the future to develop effective interventions to reduce myocardial ischemic injury and limit infarct size in patients with MI.

Dysregulation of the non-coding transcriptome (miRNAs, lncRNAs, and circRNAs) has been reported across various heart diseases, which suggests that ncRNAs are critical modulators of cardiac pathophysiology [166]. Our current knowledge regarding the origin and function of ncRNAs in the heart is still in its infancy. However, some of these ncRNAs are known to be released into the circulation and their concentration levels in blood, serum, urine, saliva, or microvesicles could allow us to discriminate between heathy and diseased subjects. For this reason, ncRNAs have gained recognition as potential non-invasive biomarkers to improve diagnosis, prognosis, and risk assessment of cardiac injury [167]. Moreover, their biochemical characteristics make them more stable, sensitive, and specific than other types of circulating biomarkers [168]. The miRNAs miR-1-5p, miR-126-5p, miR-197-5p, and miR-208a-5p have been proposed as diagnostic markers of acute MI while additional panels of ncRNAs useful in tracking complications derived from myocardial ischemia have also been reported [169,170,171,172,173]. Unfortunately, studies evaluating the true sensitivity and specificity of these ncRNAs as biomarkers in different populations are still lacking. Additionally, mimicking opioid cardioprotection through in vitro delivery of ncRNA as drug targets represents a promising therapeutic strategy for a myriad of cardiac conditions [174]. However, there is still not enough evidence on the effectiveness of these new approaches in CVD treatment and many challenges remain such as specific delivery, poor cellular uptake, off-target effects, and immunogenicity [175].

Opioids are drugs with a narrow therapeutic index that are commonly used to treat pain. Multiple genetic factors regulate opioid pharmacokinetics and pharmacodynamics [176,177]. The pharmacokinetic genes coding for phase I and II enzymes involved in opioid metabolism are highly polymorphic [176,177]. Thus, the presence of single-nucleotide polymorphisms, copy number variations, or differences in expression levels of pharmacokinetic genes can manifest as variations of opioid-induced responses among different individuals. The field of pharmacogenomics has mostly focused on drug targets and its relationship with metabolism or transport genes. Since ncRNAs can potentially regulate the expression of pharmacogenomic-related genes and processes, they could have profound implications on personalized responses to opioid analgesics.

## Figures and Tables

**Table 1:** Effect of opioids on miRNA expression in a variety of cells and tissues.

Opioid Agonist	Location	Species	miRNAs	Expression	Reference
Morphine	Macrophages	Human	miR-146a-5p miR-24-5p miR-1826-5p miR-16-5p miR-150-5p miR-30c-5p miR-155-5p miR-23a/b-5p miR-221-5p miR-15b-5p miR-132-5p	Upregulated	[109]
Morphine	Macrophages	Human	miR-26a-5p miR-191-5p miR-26b-5p miR-21-5p miR-320c-5p miR-423-5p miR-103-5p miR-107-5p miR-320a-5p miR-99b-5p miR-1915-5p miR-1469-5p miR-638-5p miR-181b-5p	Downregulated	[109]
Hydromorphone Oxycodone	Serum	Human	Let-7a miR-423-3p miR-199a-3p miR-146a/b-5p miR-23b-3p miR-24-3p miR-221-3p miR-223-3p	Upregulated	[15]
Hydromorphone Oxycodone	Serum	Human	miR-144-3p miR-451a-5p miR-192-5p miR-215-5p miR-363-3p miR-194-5p miR-140-3p miR-16-5p miR-15a-5p miR-92a-3p miR-101-3p miR-19b-3p miR-19a-3p miR-15b-3p miR-425-5p miR-93-5p miR-20a-5p	Downregulated	[15]
Morphine	Neuroblastoma derived cells	Mice	Let-7	Upregulated	[110]
Morphine	Neuronal cells	Mice	miR-23b-5p	Upregulated	[111,113]
Morphine	Embryos and neurons	Zebrafish	miR-212-5p miR-132-5p	Upregulated	[114]
Morphine Fentanyl	Hippocampus	Mice	miR-339-3p	Upregulated	[115]
Morphine	Lymphocytes	Human	miR-16-5p	Downregulated	[112]
Morphine	HEK-293 cells, Be(2)C cells and striatum	Human Mice	miR-103-5p miR-107-5p	Upregulated	[116]
Morphine	Neurons	Rat	miR-365-5p	Downregulated	[117]

**Table 2:** Effect of opioids on miRNA expression in the heart and cardiac cells.

Opioid Agonist	Location	Species	miRNAs	Expression	Reference
UFP-512 *	Heart	Rat	miR-107-3p miR-141-3p miR-350-5p	Upregulated	[146]
UFP-512 *	Heart	Rat	miR-7a/b mi-107-3p miR-200b-5p miR-376a-3p miR-134-5p	Upregulated **	[146]
Morphine	Ventricular myocytes	Rat	miR-423-5p miR-106b-5p miR-345-5p miR-3571-5p miR-130a-3p miR-133b-5p miR-3596d-5p miR-152-3p miR-15b-5p miR-3582-5p miR-374-5p miR-3596a-5p miR-3473-5p miR-3068-3p miR-16-5p miR-133a-5p miR-6215-5p	Upregulated	[132]
Morphine	Ventricular myocytes	Rat	miR-25-3p miR-466b-2-3p miR-214-3p miR-328a-5p miR-181a/b/c-5p miR-148b-3p miR-133a/b-3p miR-29a-3p let-7i miR-125a-5p miR-466b-1-3p miR-208a-3p miR-499-3p miR-466c-3p miR-483-5p miR-505-3p miR-1224-5p miR-3584-5p miR-6216-5p	Downregulated	[132]
Morphine	Cardiomyocytes ***	Rat	miR-130a-3p miR-133a/b-5p miR-16-5p miR-6215-5p miR-151-5p miR-143-3p miR-107-3p miR-125b-5p miR-150-5p miR-29a-3p miR-378b-5p	Upregulated	[133]
Morphine	Cardiomyocytes ***	Rat	Let-7i miR-1224-5p miR-125a-5p miR-133a/b-3p miR-181a-5p miR-29a-3p miR-6216-5p miR-30a/e-5p miR-6215-5p	Downregulated	[133]
Fentanyl	Cardiomyocytes	Mice	miRNA-145-5p	Upregulated	[152]
Fentanyl	Cardiomyocytes	Mice	lncRNA MALAT1	Downregulated	[152]

* DOR agonist. ** Under hypoxic conditions. *** Derived from sham or failing hearts. MALAT1: Metastasis-associated lung adenocarcinoma transcript 1.
